# Case report: Pathological complete response of pregnancy associated pulmonary enteric adenocarcinoma to chemoradiotherapy

**DOI:** 10.3389/fonc.2024.1290757

**Published:** 2024-02-23

**Authors:** Yukiko Nemoto, Koji Kuroda, Rintaro Oyama, Masataka Mori, Shohei Shimajiri, Fumihiro Tanaka

**Affiliations:** ^1^ Second Department of Surgery (Chest Surgery), School of Medicine, University of Occupational and Environmental Health, Kitakyushu, Fukuoka, Japan; ^2^ Department of Pathology and Cell Biology, School of Medicine, University of Occupational and Environmental Health, Kitakyushu, Fukuoka, Japan

**Keywords:** pulmonary enteric adenocarcinoma, pregnancy-associated adenocarcinoma, brain metastasis, chemoradiotherapy, complete pathological response, case report

## Abstract

Pulmonary enteric adenocarcinoma (PEAC) is a rare lung adenocarcinoma with morphological features similar to those of primary and metastatic colorectal adenocarcinoma. To date, only a few studies have reported the therapeutic effects of chemoradiotherapy on PEAC. This report describes the case of a 28-year-old woman with pregnancy-related PEAC who presented with left shoulder pain. A superior sulcus tumor was identified in the left thoracic cavity, and the biopsy indicated more than 50% intestinal differentiation components. Moreover, immunohistochemical staining revealed positive CDX2 and CK7 expression. Positron emission tomography-computed tomography, upper endoscopy, colonoscopy, and small intestinal capsule endoscopy revealed no gastrointestinal malignancies. The patient was diagnosed with locally advanced PEAC (clinical stage T4N0M0; stage IIIA). Therefore, the patient was treated with preoperative chemoradiotherapy and underwent gross total resection during surgery. Pathological evaluation of the specimen revealed no residual tumor, indicating that the chemoradiotherapy for PEAC was highly effective. One subsequent brain metastasis was also resected, and the patient has not experienced recurrence in 28 months since this resection and continues to be monitored regularly. This is the first pathologically confirmed report of the use of chemoradiotherapy (carboplatin [CBDCA] and paclitaxel [PTX]) for PEAC and its clinical efficacy. Unlike previous reports, the efficacy of this treatment is attributed to the use of PTX in preoperative chemotherapy and the p21− status of the patient, which may have increased sensitivity to chemoradiation therapy. Therefore, chemoradiotherapy (CBDCA + PTX) may be a viable treatment option for advanced intestinal lung adenocarcinoma.

## 1 Introduction

Primary pulmonary enteric adenocarcinoma (PEAC), which was first reported in 1991, is a rare, invasive adenocarcinoma morphologically similar to colorectal adenocarcinoma (1). According to the 2015 WHO classification, the diagnostic criteria for PEAC stipulate that over 50% of adenocarcinomas should display an intestinal differentiation component, with the clinical exclusion of an enteric primary within the gastrointestinal tract. Moreover, immunohistochemistry analysis indicates that some enteric adenocarcinoma tumors express CDX2, CK20, and CK7 (2). PEAC is regarded as a rare subtype of lung adenocarcinoma with an overall prevalence of 0.5% in non-small cell lung cancer (NSCLC) (3). Currently, its treatment is similar to that of NSCLC, and no specific treatment plan has been established. Moreover, only a few studies have described the effectiveness of radiation chemotherapy treatment.

Herein, we present the case of a 28-year-old pregnant woman who presented with primary PEAC and superior sulcus tumor (SST). After delivery, the patient underwent radiation chemotherapy, which resulted in a 39% tumor reduction. We surgically removed the primary tumor, pathologically confirmed the complete disappearance of the tumor cells, and immunohistochemically ruled out the presence of isolated malignant cells.

## 2 Case description

**Figure 1:**
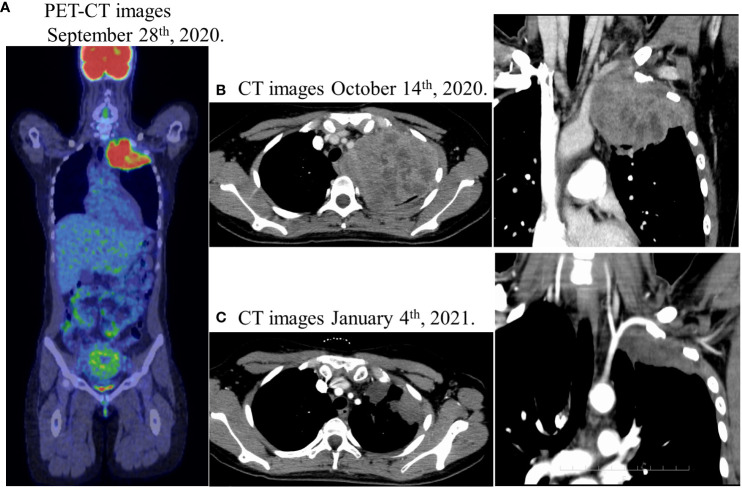
**(A)** Positron emission tomography-computed tomography (PET-CT) showed an abnormal FDG accumulation in the left pulmonary apex (SUVmax 12.58), no abnormal FDG accumulation in the gastrointestinal tract, and no suspicious findings of distant organ or lymph node metastasis. **(B)** Lung enhanced computed-tomography (CT) scan showed a massive soft tissue shadow, about 107 × 88 × 82 mm in the upper lobe of the left lung on October 14th. **(C)** Lung enhanced CT scan showed a soft tissue shadow, about 72 × 50 × 42 mm in the upper lobe of the left lung on January 4th after chemoradiotherapy, indicating a partial response with a reduction of 40%.

The patient was a 28-year-old woman who had complained of left shoulder pain since January 2020 and had a positive pregnancy test in May of the same year. The pregnancy progressed well. However, she experienced left shoulder pain that worsened gradually, drooping of the left eyelid, decreased sweating on the left side of her face, and an abnormal sensation in the left arm. The patient had a 10-year history of smoking 10 cigarettes per day since the age of 18 years. In August 2020 (28 weeks of gestation), chest magnetic resonance imaging (MRI) revealed a mass in the left upper lobe. She delivered via cesarean section in September 2020 (30 weeks of gestation). Computed tomography (CT) performed after delivery revealed a 107 × 88 × 82 mm mass in the left pulmonary apex. The tumor was an SST with a suspected invasion of the dorsal first to fourth ribs and the subclavian artery. We observed no evidence of lymph node enlargement (**Figure 1**). Tumor markers were high, with carcinoembryonic antigen (CEA) at 10.8 ng/mL, CYFRA at 7.2 ng/mL, and SLX at 45.4 U/mL, whereas CA19-9, SCC, and NSE were normal.

**Figure 2:**
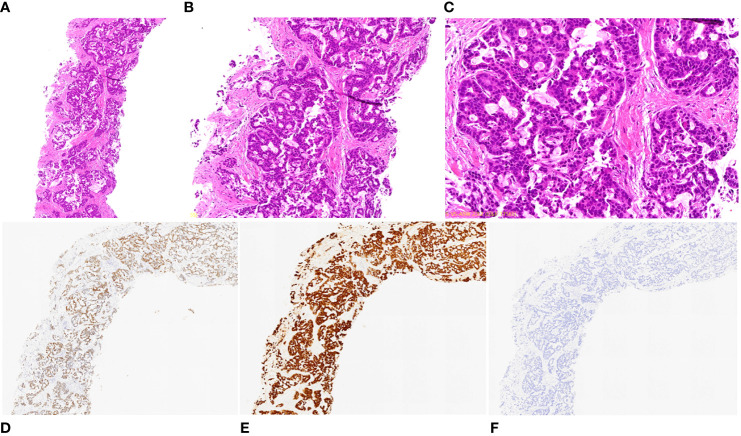
Pathological and immunohistochemical examination results of lung biopsy. **(A)** hematoxylin-eosin staining (HE), magnification ×40; **(B)** HE, magnification ×100; **(C)** HE, magnification ×200; **(D)** CDX-2 positivity, magnification ×40; **(E)** CK7 positivity, magnification ×40; **(F)** CK20 negativity, magnification ×40.

**Figure 3:**
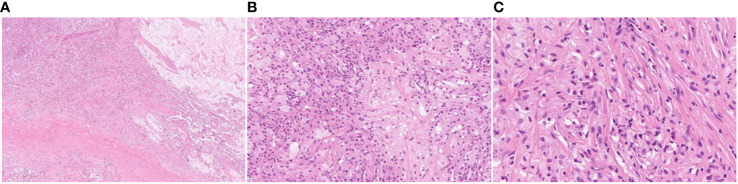
Pathological examination results after lung cancer surgery. The nodular lesion beneath the inflamed lung is composed of almost completely necrotic tissue without evidence of viable atypical or carcinoma cells. The pathology results revealed no residual tumor (pathological complete response, Ef3). **(A)** HE, magnification ×20; **(B)** HE, magnification ×100; **(C)** HE, magnification ×200.

Percutaneous lung biopsy revealed an adenocarcinoma upon pathological examination, with a cribriform arrangement of atypical cells with darkly stained enlarged nuclei, eosinophilic reticulum, and a fused tubular structure. Immunostaining was positive for CK7 and CDX-2, whereas it was negative for TTF-1, Napsin, CK20, ER, PgR, and GCDFP15 (**Figure 2**). For genetic analysis, we conducted both the cancer gene panel using Amoy 9in1 plus by SRL and Next-Generation Sequencing (NGS) using the ION Torrent Genexus System (Thermo Fisher Scientific, Waltham, MA, USA) on the biopsy sample. Amoy 9in1 plus is designed to identify somatic variations in 9 cancer-related genes, while the ION Torrent Genexus system is designed to identify somatic variations in 50 cancer-related genes, including *EGFR*, *ALK*, *KRAS*, *NRAS*, *BRAF*, *RET*, *MET*, and *NTRK*. None of these genes exhibited any detectable mutations in this case. A systemic examination, including MRI of the head, CT scan of the chest and abdomen, positron emission tomography-computed tomography scan, upper and lower gastrointestinal endoscopy, and capsule endoscopy of the small intestine, revealed no neoplastic lesions, except in the lungs (**Figure 1A**). Therefore, the patient was diagnosed with locally advanced primary PEAC stage IIIA (T4N0M0) with chest wall invasion of the lung apex. The patient was treated with five courses of weekly carboplatin + paclitaxel (CBDCA + PTX) and 66 Gy/33 Fr of preoperative chemoradiotherapy in October 2020. A chest CT performed in January 2021 indicated a partial response with a reduction of 40% (**Figures 1B, C**), and tumor markers improved to the normal range with CEA 1.9 ng/mL, CYFRA 1.1 ng/mL, and SLX 33.3 U/mL. In January 2021, the patient underwent left upper lobectomy (ND2a-1), combined 1st–4th rib resection reconstruction, and Th1 combined resection. Complete gross resection of the tumor was performed. The pathology results revealed no residual tumor (pathological complete response, Ef3) (**Figure 3**) or lymph node metastasis. Immunohistochemically, atypical cells expressing CEA or cytokeratin, such as CAM5.2 and AE1/AE3, were absent. The pathology indicated a significant response to preoperative chemoradiotherapy. The sampling was conducted according to the Recommendations of the IASLC (4). We observed no postoperative complications, and the patient was transferred to the hospital for rehabilitation on postoperative day 19.

**Figure 4:**
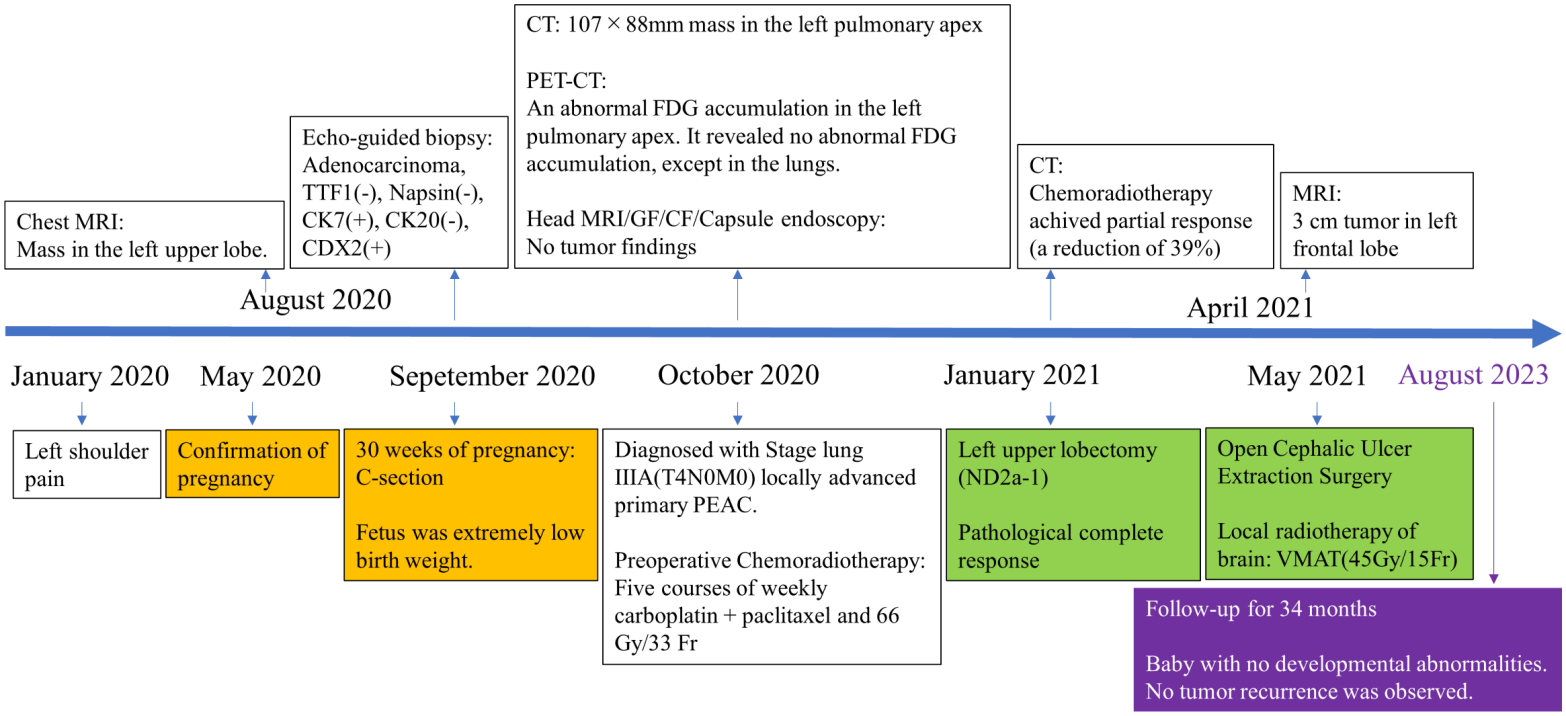
Timeline of clinical events from symptom onset to last follow-up appointment. (CT, computed-tomography; MRI, magnetic resonance imaging; PET-CT, positron emission tomography-computed tomography; GF, gastrofiberscopy; CF, colonofiberscopy).

However, in April 2021, she developed weakness in her right upper extremity. Head MRI revealed a 3 cm mass in the cortical to the subcortical white matter of the left frontal lobe, but no re-elevation of tumor markers or other metastatic sites was observed. Therefore, we suspected a metastatic brain tumor. Left frontal craniotomy and tumor removal were performed, and postoperative irradiation therapy with 45 Gy/15 Fr was administered to the brain metastasis site. The pathological results were consistent with metastasis from lung cancer, as it was an adenocarcinoma with invasive growth-forming snoring fused tubular adenoid ducts. As with the biopsy specimens, immunostaining was positive for CDX-2, p53, CK7 (focal), and CK20 (focal) and negative for TTF-1, PD-L1 (IHC 22C3), and p21, with a Tumor Proportion Score (TPS) of <1% (**Supplementary Figure S1**). The fetus was delivered at extremely low birth weight, but is growing healthily. After surgery for the metastatic brain tumor, we proposed systemic postoperative adjuvant chemotherapy, but the patient continues to be followed up at her request. As of August 2023, no tumor recurrence was observed. The treatment history of this patient is shown in **Figure 4**.

## 3 Discussion

In our case study, the preoperative biopsy of the primary lung tumor revealed atypical fused tubular structures in over 50% of the specimens, which were CK7- and CDX-2-positive. Cribriform and fused ducts appear in moderately differentiated tubular adenocarcinomas in colorectal cancer (5), suggesting intestinal differentiation of lung adenocarcinoma. Furthermore, the samples were positive for CK7 and CDX-2, and the differential diagnosis between PEAC and colorectal adenocarcinoma had a sensitivity of 71.3% and specificity of 82% (6). We diagnosed this case as PEAC because over 50% of the lung adenocarcinomas presented fused tubular structures. The tumor was positive for CDX2 and CK7; tumors were not identified elsewhere except in the lungs. Gene mutations in *KRAS* (47.1 ± 33.7%), *EGFR* (12.4 ± 15.7%), *BRAF* (2.6 ± 6.3%), and *ALK* (5.1 ± 7.1%) (7) are reportedly associated with PEAC. There have been reports of the identification of rare *EGFR exon 19 P753S* gene mutation by using NGS for genetic mutation analysis in PEAC (8), and NGS is recommended for monitoring oncogene mutations. In this case, NGS was also employed for gene mutation analysis; however, no mutations were detected among the 50 genes associated with the tumor. Gong et al. (9) reported in their review that the average age of patients with PEAC was 50–60 years, with no sex-related differences. The patients experienced disease onset at a young age, and approximately 50% were smokers.

This case was diagnosed as PEAC and superior sulcus tumor (SST). Radiotherapy is not typically recommended as a routine treatment for PEAC. However, we made the decision to administer preoperative chemoradiotherapy because the Southwest Oncology Group 9416 trial (10) and the Japan Clinical Oncology Group trial 9806 (11) have both provided evidence supporting the efficacy of preoperative chemoradiotherapy for SST. In this case, preoperative chemoradiotherapy resulted in a tumor shrinkage of 39%, according to the RECIST (12) evaluation. Pathological evaluation of the surgical specimen revealed no tumor components (Ef3), and preoperative chemotherapy was considerably effective. Zhao et al. (3) evaluated 24 patients with PEAC who underwent surgery and reported that 13 patients with Stage I cancer survived, with the longest survival being > 31 months after surgery. Stage II cancer was observed in four patients, one of whom died 20 months post-surgery; nonetheless, the remaining patients were alive and survived for at least 30 months post-surgery. Stage III cancer was present in six patients, four died at 1–19 months post-surgery, and two survived for over 29 months. One patient with Stage IV cancer underwent surgery and survived for > 21 months. These findings collectively suggest that surgery is the most effective treatment for early-stage PEAC.

Although a few reports exist on the response to chemotherapy (13–16), this treatment regimen is generally considered unsuccessful (9). Notably, Tu et al. (14) provided the only report on PEAC treated with chemoradiotherapy, and observed a partial response after four courses of pemetrexed (PEM) + cisplatin and 30 irradiation treatments. However, there is a of lack reports on Ef3 response in pathological specimens after radiation chemotherapy, such as in this case.

Referring the cohort study of colon cancer with histological characteristics similar to those of PEAC by Nosuke A et al. (17), which showed that colon cancer displaying p21-negative and p53-positive immunostaining, exhibited extended overall survival after chemotherapy. Based on this result, we conducted p21 (monoclonal anti-p21WAF1/CIP1 antibody [Clone EA10]) and p53 (monoclonal anti-p53 antibody [Clone DO-7]) immunostaining on the brain metastasis specimen in this case to explore the underlying factors contributing to the remarkable response to preoperative radiotherapy and chemotherapy; this case was also p21-negative and p53-positive (**Supplementary Figure S2**). While the aforementioned cohort study did not elucidate the specific mechanisms by which p21 or p53 might influence chemotherapy outcomes, we delved into the existing literature to gain further insights into the effects of radiotherapy and chemotherapy on tumors with a similar p21-negative and p53-positive profile.

The p53 protein, a product of *TP53*, is a transcription factor distributed in the nucleus and cytoplasm and binds specifically to DNA to activate the expression of multiple target genes. Normally, p53 is ubiquitinated by ubiquitin ligases, such as MDM2, and is immediately degraded. However, inhibition of ubiquitination and increase in intracellular p53 protein levels owing to various cellular stresses, such as DNA damage, lead to its activation and stabilization through post-translational modifications, such as phosphorylation and acetylation. This results in the sequence-specific transcriptional activation of multiple downstream genes, such as *WAF1/CIP1*. p53 is involved in cell-cycle checkpoint mechanisms, including G2/M and G1/S phase arrest. It is also involved in the induction of tumor cell death via apoptosis, DNA repair, and other crucial cellular functions (18). Transcriptional activation of *p21WAF1* is crucial for suppressing cell proliferation (17, 18). p21 is a reversible inhibitor of cell-cycle progression. The induction of p53-dependent *p21WAF1* in response to DNA damage leads to irreversible G1 or G2 phase arrest, resulting in apoptosis. In contrast, p21 is expressed in a p53-independent manner via a different pathway (19).

Alterations in p21 have also been suggested to affect the sensitivity of cancers to chemotherapy and radiation therapy, with some reports indicating that p21-deficient cell lines exhibit enhanced sensitivity to radiation-induced apoptosis in vitro (20, 21). Similar to this case, cancer cells treated with microtubule inhibitors also exhibit increased apoptosis and cell death when p21 is depleted (22). These reports leading us to hypothesize that the absence of p21 could be a key factor contributing to the substantial response to radiotherapy and chemotherapy in this case.

Conversely, tumors with p53 mutations are generally considered to have decreased sensitivity to most anticancer drugs (e.g. alkylating agents, platinum-based drugs, antimetabolites, and topoisomerase inhibitors) compared to tumors with wild-type p53 (23). Therefore, we did not consider p53 positivity as the primary cause of the remarkable response to chemotherapy in this instance. However, paclitaxel and other anticancer agents that inhibit microtubule polymerization or depolymerization reportedly do not change susceptibility with or without *TP53* mutation (23). Paclitaxel is also more effective against mutant p53 cells (24). In vivo studies have reported that paclitaxel releases TNF-α, a cytokine released by macrophages, causing p53-independent apoptosis (25). We considered that these reports are the reason why the chemotherapy of Paclitaxel was successful in this case despite the positive p53.

Although p53 and p21 have been studied in many cancers for their sensitivity to chemoradiotherapy and prognosis, their diverse expression pathways may involve many factors, and further studies are warranted to elucidate their molecular mechanisms.

In this case, the brain tumor was discovered postoperatively, and the pathological examination of the resected brain tumor confirmed it as a metastasis originating from PEAC. While distant metastases to the lung, bone, liver, distant lymph nodes, and adrenal glands have been reported in advanced stages of PEAC (26), to the best of our knowledge, this case represents the first documented instance of brain metastasis in PEAC.

Furthermore, this case was regarded as pregnancy-related lung cancer owing to its diagnosis during pregnancy (27). According to a 2021 review of pregnancy-associated lung cancer (9), 11 of 1063 women with lung cancer at the Guangdong Lung Cancer Institute had pregnancy-associated lung cancer. Of the 77 reported cases of pregnancy-associated lung cancer, 52 (68%) were lung adenocarcinomas, 11 (14%) were treated with surgery, 22 (29%) with radiation, 33 (42.8%) with chemotherapy, and 30 (39%) with molecular targeted therapy. Fifty-four (70%) began treatment after delivery (28). To the best of our knowledge, this is the first report on PEAC in pregnancy-related lung cancer.

In this case, preoperative chemoradiotherapy proved to be pathologically successful in a 28-year-old pregnant woman with PEAC involving the pulmonary apex of the chest wall. Subsequently, a single brain metastasis identified in the early postoperative period was resected, and the patient survived without recurrence for 32 months after surgery. Thus, our findings suggest that preoperative chemoradiation with CBDCA + PTX + radiotherapy may be effective for advanced PEAC. However, owing to the limited case reports on radiation chemotherapy for PEAC, further research is warranted.

## Data availability statement

The original contributions presented in the study are included in the article/**Supplementary Materials**. Further inquiries can be directed to the corresponding author.

## Ethics statement

The studies involving humans were approved by The institutional review board of the University of Occupational and Environmental Health, Japan. The studies were conducted in accordance with the local legislation and institutional requirements. The participants provided their written informed consent to participate in this study. Written informed consent was obtained from the individual(s) for the publication of any potentially identifiable images or data included in this article.

## Author contributions

YN: Conceptualization, Data curation, Formal analysis, Investigation, Visualization, Writing – original draft, Methodology. KK: Writing – review & editing, Data curation, Supervision. RO: Investigation, Methodology, Writing – review & editing. MM: Conceptualization, Investigation, Writing – review & editing. SS: Conceptualization, Data curation, Formal analysis, Investigation, Methodology, Supervision, Writing – review & editing. FT: Supervision, Writing – review & editing.

## Supplementary material

Supplementary Figure S1 Histopathological and immunohistochemical findings of brain metastatic tumor. The pathological results were consistent with metastasis from lung cancer, as it was an adenocarcinoma with invasive growth-forming snoring fused tubular adenoid ducts. **(A)** Hematoxylin-eosin staining (HE), magnification ×10; **(B)** HE, magnification ×100; **(C)** HE, magnification ×200; **(D)** CDX-2 positivity, magnification ×50; **(E)** CK7 positivity (focal), magnification ×50; **(F)** CK20 positivity (focal), magnification ×50.

Supplementary Figure S2 Immunohistochemical findings of brain metastatic tumor. **(A)** p53 positivity, magnification ×100; **(B)** p21 negativity, magnification ×100.
