# Supplementary figures and images for: Case report: Pathological complete response of pregnancy associated pulmonary enteric adenocarcinoma to chemoradiotherapy
Source: Front Oncol. 2024 Feb 23;14:1290757. doi: 10.3389/fonc.2024.1290757 (PMC10924307; doi:10.3389/fonc.2024.1290757)

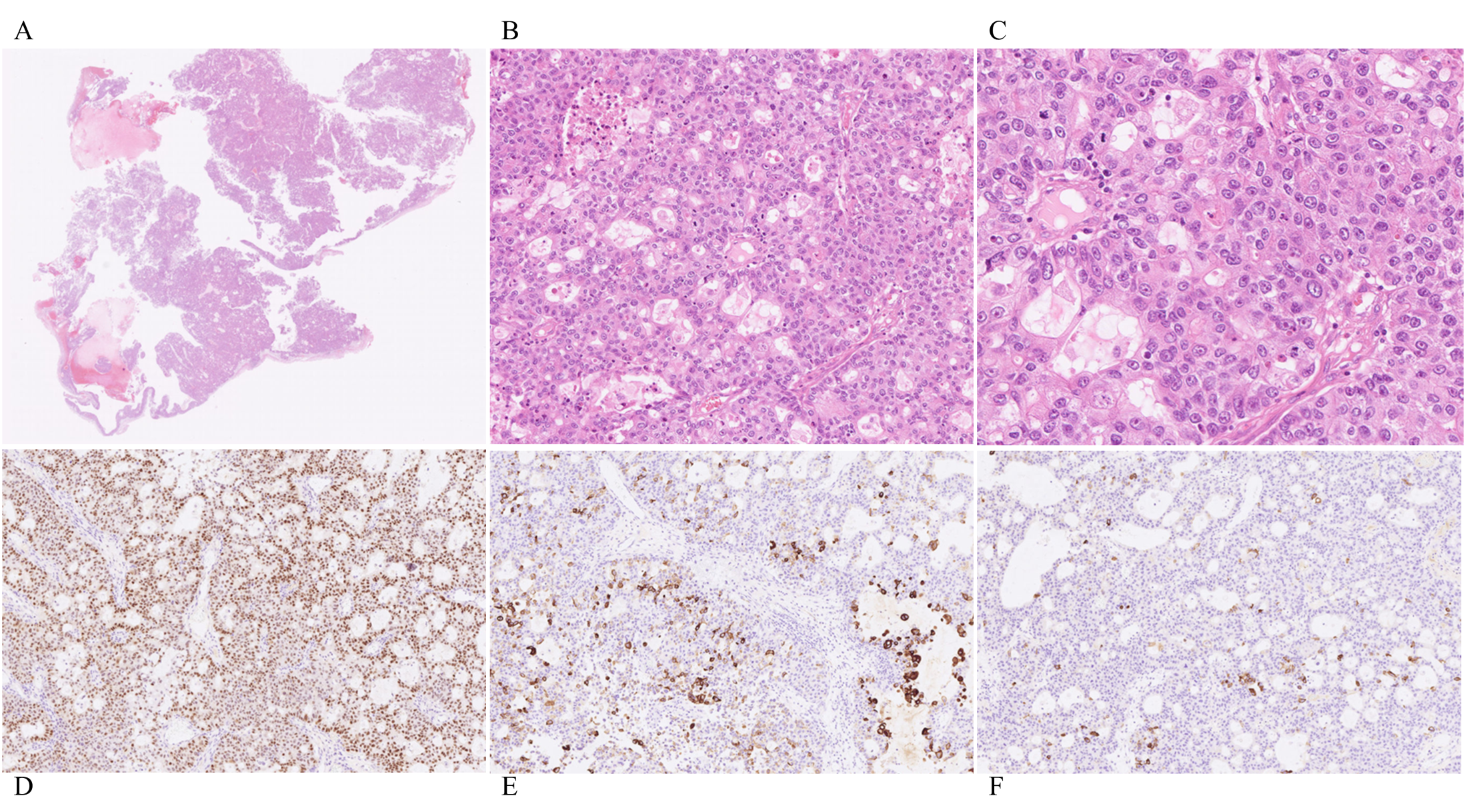

Supplement: Supplementary Figure S1 — Histopathological and immunohistochemical findings of brain metastatic tumor. The pathological results were consistent with metastasis from lung cancer, as it was an adenocarcinoma with invasive growth-forming snoring fused tubular adenoid ducts. (A) Hematoxylin-eosin staining (HE), magnification ×10; (B) HE, magnification ×100; (C) HE, magnification ×200; (D) CDX-2 positivity, magnification ×50; (E) CK7 positivity (focal), magnification ×50; (F) CK20 positivity (focal), magnification ×50. [file Image_1.tif]

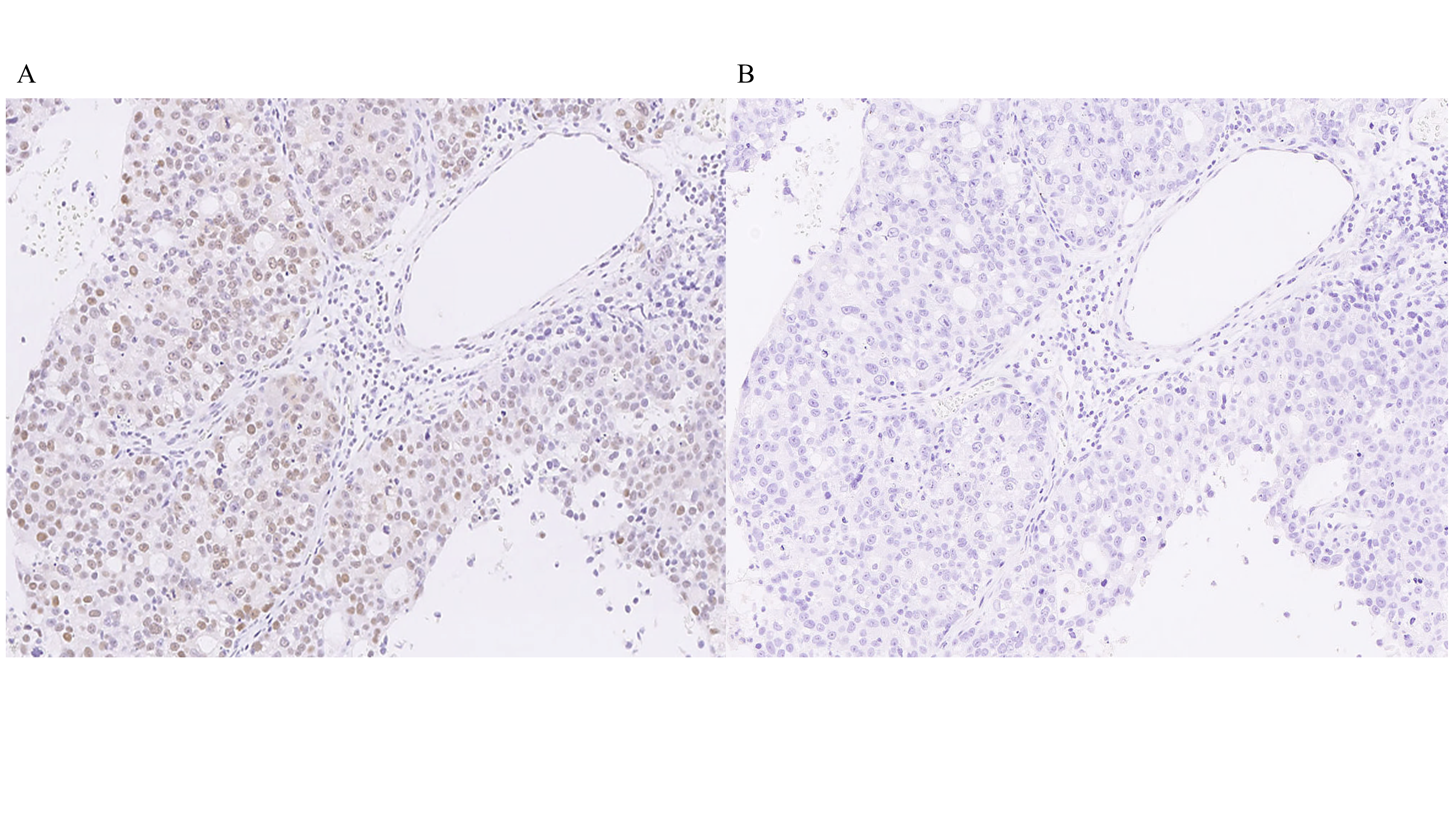

Supplement: Supplementary Figure S2 — Immunohistochemical findings of brain metastatic tumor. (A) p53 positivity, magnification ×100; (B) p21 negativity, magnification ×100. [file Image_2.tif]
